# Utilization and Effectiveness of a Message-Based Tobacco Cessation Program (mCessation) in the Chinese General Population: Longitudinal, Real-world Study

**DOI:** 10.2196/44840

**Published:** 2023-05-02

**Authors:** Zheng Su, Xiaowen Wei, Anqi Cheng, Xinmei Zhou, Jinxuan Li, Rui Qin, Yi Liu, Xin Xia, Qingqing Song, Zhao Liu, Liang Zhao, Dan Xiao, Chen Wang

**Affiliations:** 1 Department of Tobacco Control and Prevention of Respiratory Diseases China-Japan Friendship Hospital Center of Respiratory Medicine Beijing China; 2 World Health Organization Collaborating Center for Tobacco Cessation and Respiratory Diseases Prevention Beijing China; 3 National Clinical Research Center for Respiratory Diseases Beijing China; 4 Institute of Respiratory Medicine Chinese Academy of Medical Sciences Beijing China; 5 National Center for Respiratory Medicine Beijing China; 6 Peking Union Medical College Chinese Academy of Medical Sciences Beijing China; 7 Capital Medical University China-Japan Friendship School of Clinical Medicine Beijing China

**Keywords:** smoking cessation, real-world evidence, text message, general population

## Abstract

**Background:**

Randomized controlled trials on text message interventions for smoking cessation have shown they are effective and recommended for tobacco control. However, the effectiveness in real-world settings is largely unknown, especially in low- and middle-income countries.

**Objective:**

This study aimed to provide real-world evidence about the utilization and effectiveness of a message-based tobacco cessation program (mCessation) in China.

**Methods:**

From May 2021 to September 2022, 16,746 people from the general population participated in the mCessation program provided by the World Health Organization. All participants received text messages on smoking cessation via instant messaging for 6 months, and they were also required to report smoking status. We randomly selected 2500 participants and interviewed them by telephone to determine the 7-day point prevalence abstinence rate at 6 months. Descriptive statistics were used to analyze population characteristics and abstinence rate. Logistic regression analysis was performed to explore risk factors for the abstinence rate.

**Results:**

Among the 2500 participants, the mean age was 35 years, and most (2407/2500, 96.20%) were male. The prevalence of tobacco dependence and light degree of tobacco dependence were 85.70% (2142/2500) and 89.10% (2228/2500), respectively. For respondents (953/2500, 38.10%), the 7-day point prevalence abstinence rate at 6 months was 21.90% (209/953). Participants older than 40 years or with tobacco dependence had significantly higher abstinence rates than those who were younger than 30 years old (odds ratio [OR] 1.77, 95% CI 1.06-3.29) or without dependence (OR 1.64, 95% CI 1.08-2.51), respectively. However, married people or heavily dependent smokers tended to find it more difficult to successfully quit smoking compared with unmarried people (OR 0.57, 95% CI 0.34-0.93) or lightly dependent smokers (OR 0.16, 95% CI 0.02-0.98), respectively.

**Conclusions:**

In a real-world setting, mCessation China was generally acceptable to men and lightly dependent smokers, and it could help 1 in 5 smokers aged 18 years to 67 years quit smoking. However, strategies to increase awareness of young and married adults may improve implementation and abstinence rates.

## Introduction

Tobacco use is a common and preventable risk factor for a wide range of chronic, noncommunicable diseases [1]. China represents approximately 40% of the world’s total cigarette use [2], causing about 20% of all adult male deaths in 2010 [3]. According to the China Adult Tobacco Survey in 2018, there were 308 million smokers, and a relatively low percentage (16.2%) intended to quit smoking within 12 months [4]. This indicates that tens of millions of smokers needed help from medical and health institutions such as smoking cessation clinics, because most self-help quit attempts failed [5]. However, our previous nationwide survey of available smoking cessation support programs showed an imbalanced distribution of resources, such as smoking cessation clinics, and limited availability of medications in China [6]. Therefore, tobacco cessation interventions that are of low cost, are convenient, and have potential for wide reach are urgently needed in low- and middle-income countries (LMICs).

Text message interventions for smoking cessation have been proven to be efficacious and have great potential for serving geographically and economically diverse populations. On the one hand, a large number of randomized controlled trials (RCTs) have shown that text-based tobacco cessation programs can significantly increase smoking abstinence rates. However, the definition of the primary outcome of smoking abstinence in previous studies has varied, for example, biochemically verified continuous abstinence at 6 months in studies in the United Kingdom [7], the United States [8], Hong Kong [9,10], and mainland China [11]; self-reported, 7-day point prevalence abstinence (PPA) at 6 months in Norway and Hong Kong [12,13]; 1-month PPA in Sweden [14]; and self-reported, 30-day PPA at 9 months in the United States [15]. On the other hand, a mobile intervention could be achieved through WeChat (a popular, multipurpose messaging, social media, and mobile payment app). There are more than 1.1 billion WeChat users in China [16]. Therefore, the intervention could offer an opportunity to extend the reach of tobacco cessation interventions outside of traditional health care settings via a mobile phone app that most adults already own, is generally always on, and is already used many times each day.

However, the real-world effectiveness of text messaging programs is largely unknown, although great efficacy had been achieved in clinical trials [7-15]. Evaluation of effectiveness in real-world settings is of great significance, because the public health impact of an intervention crucially depends on not only its efficacy but also real-world uptake, which could be influenced by population-specific characteristics and other factors [17]. To our knowledge, only 3 previous studies explored the real-world effectiveness of a text messaging intervention [18-20]. Primary outcomes in 2 studies of smoking cessation by the Veterans Health Administration were short-term abstinence at 5 weeks [18] or self-reported 30-day PPA at 6 months [20]. In a program conducted in India, self-reported 30-day PPA at 4 months to 6 months was used [21]. However, no published studies have explored the effectiveness of self-reported 7-day PPA at 6 months as the primary outcome, even though this abstinence rate was commonly used in previous RCTs.

Therefore, to gain a more comprehensive understanding of the effectiveness of a text message intervention, we conducted a real-world analysis of 2 years of nationwide data from a message-based tobacco cessation (mCessation) program. The mCessation program is based on a library of 162 short text messages provided by the World Health Organization (WHO) and can be used by scanning a WeChat QR code. In this study, we aimed to provide real-world data about the utilization and effectiveness of the mCessation program in China.

## Methods

### Study Population

**Figure 1:**
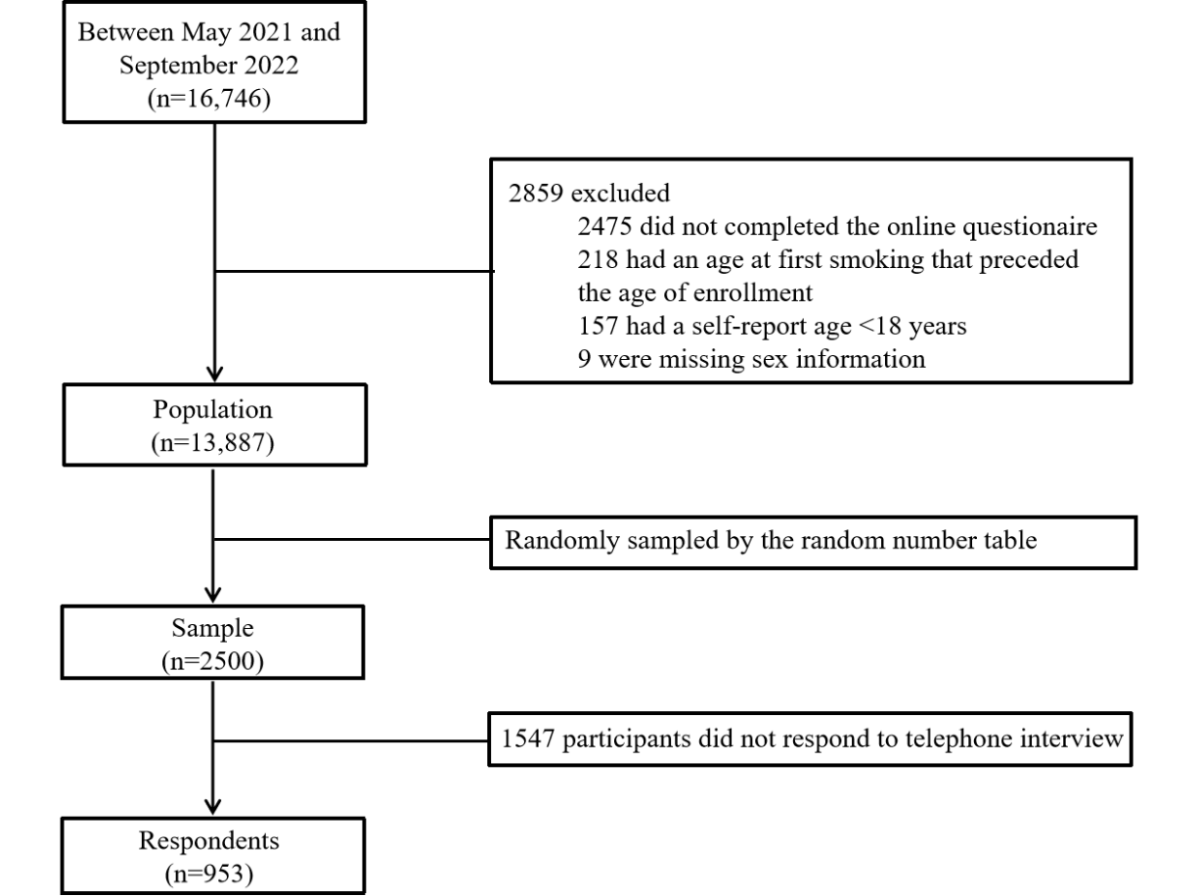
Flowchart of the selection of mCessation program participants.

Between May 2021 and September 2022, the mCessation program was disseminated nationwide through the following channels: (1) recommendations from health care providers, (2) special publicity activities, (3) social media posts including videos, (4) posters in hospitals, (5) WeChat official accounts. A total of 16,746 people from the general population participated in the mCessation program. Of these, 14,271 participants completed the online questionnaire and were considered successful subscribers. Then, 218 were excluded due to an age when first started smoking that preceded the age of enrollment, 157 subscribers were excluded due to a self-reported age lower than 18 years, and 9 subscribers were excluded due to missing sex information. Of the remaining 13,887 subjects, 2500 individuals were randomly sampled by the random number table and were interviewed by telephone. Finally, only 38.1% (953/2500) of the respondents were interviewed to determine the 7-day PPA rate at 6 months. The flowchart is provided in Figure 1.

### Message-Based Tobacco Cessation (mCessation) Program and Follow-up Telephone Interview

The mCessation program, as part of the “Be Healthy Be Mobile” initiative by the WHO and International Telecommunication Union using mobile technology to combat noncommunicable diseases [22], was a preprogrammed library of 162 short text messages. Subscribers were required to provided demographic information, age when they started/ended smoking, number of cigarettes smoked per day, smoking dependence, dependence degree, time when they smoked the first cigarette after waking, and abstinence reasons at enrollment. Then, each user was required to set a quit date in order to receive text messages containing tips and encouragement to quit smoking and assessment questions to evaluate users’ cravings, mood, and abstinence. Daily messages began 2 weeks prior to the quit date and ended 42 days after the quit date; then, an additional abstinence assessment question (“Have you quit smoking or continued to smoke?”) was asked 3 times, at 1 month, 3 months, and 6 months after the quit date. In addition, all participants were interviewed by telephone to determine the 7-day PPA rate at 6 months (“Have you quit smoking within the past 7 days?”).

### Diagnosis of Tobacco Dependence

The diagnostic criteria for tobacco dependence were based on international criteria (International Classification of Diseases [ICD-10] [23], DSM-5 [24]) and tailored to the Chinese population according to the China Clinical Guideline for Tobacco Cessation (2015 version) [25]. A 6-item Chinese version of the Fagerström Test for Nicotine Dependence (FTND) scale [26] (Table S1 in Multimedia Appendix 1) was administered, with the total score ranging from 0 to 10 and a higher score representing heavier dependence. Tobacco dependence was diagnosed if they had 3 or more of the following 6 symptoms or signs: (1) craving or a strong desire or urge to use tobacco; (2) a persistent desire or unsuccessful efforts to cut down or control tobacco use; (3) experiencing tobacco withdrawal symptoms (such as irritability, frustration, anger, anxiety, difficulty concentrating, increased appetite, restlessness, insomnia) after abrupt cessation of tobacco use or reduction in the amount of tobacco used; (4) tolerance, defined as the need for markedly increased amounts of tobacco to achieve the desired effect; (5) given up or reduced important social, occupational, or recreational activities because of tobacco use; and (6) continued tobacco use despite knowledge of having a persistent or recurrent physical or psychological problem that is likely to have been caused or exacerbated by tobacco. Individuals were divided into light (0-3 points), moderate (4-6 points), and severe (≥7 points) tobacco dependence according to the FTND scores.

### Data Analysis

In this real-world analysis, descriptive statistics were used to calculate percentages for qualitative data or the mean and SD for quantitative data. Among 2500 interviewees, we analyzed the abstinence rates at 7 days, 1 month, 3 months, and 6 months after the quit date of both respondents and nonrespondents. Of all respondents, the 7-day PPA rate at 180 days was calculated, and multivariate logistic regression analysis including demographic variables and smoking questions was performed to explore influencing factors for the abstinence rate. In addition, the influencing factors for not responding were also analyzed, and sensitivity analysis was conducted to compare baseline characteristics between respondents and nonrespondents to the telephone interview. In the logistic regression models, odds ratios (ORs) and 95% CIs were calculated, and *P*<.05 was considered statistically significant for all tests. All statistical analyses were done with SAS 9.4 (SAS Institute Inc).

### Ethical Considerations

The study was approved by the institutional review board of the China-Japan Friendship Hospital (2022-KY-183). This study complied with the Declaration of Helsinki. Informed consent was obtained from each participant. Study data from the individuals are anonymous, and there was no compensation for all the participants.

## Results

### Characteristics of Subscribers to the mCessation Program

Demographic characteristics and baseline smoking information for all participants are shown in Table 1. Among 2500 participants, the mean age was 35.10 (SD 9.88) years, and 96.2% (2407/2500) were male. The description of the demographic characteristics revealed that mCessation subscribers were more likely to be married, have a tertiary education, and work in enterprises. The main channel for accessing the program was social media posts. Nearly all the participants (2469/2500, 99.1%) were cigarette users, and the average pack-years and age when they started smoking were 15.56 (SD 13.92) years and 17.94 (SD 4.50) years, respectively. In general, current smokers were dependent on tobacco, and the degree of tobacco dependence was light. More than two-thirds (1738/2500, 69.5%) of individuals attended the mCessation program because they were worried about the hazardous effect of tobacco on their children or wanted to prevent diseases.

**Table 1:** Demographic characteristics and smoking profiles of the participants (n=2500).

Variables		Results
Age (years), mean (SD)		35.10 (9.88)
**Age (years), n (%)**		
	<30	746 (29.8)
	30 to <40	1002 (40.1)
	≥40	752 (30.1)
**Gender, n (%)**		
	Male	2407 (96.2)
	Female	93 (3.7)
**Education level, n (%)**		
	Primary school or below	59 (2.4)
	Middle school	678 (27.1)
	College/university or above	1761 (70.5)
**Employment status, n (%)**		
	Unemployed	352 (14.1)
	Working in a public service unit	697 (27.9)
	Working in an enterprise	1448 (58.0)
**Marital status, n (%)**		
	Unmarried	645 (25.8)
	Married	1707 (68.4)
	Divorced	144 (5.8)
**Information source, n (%)**		
	Recommended by health care providers	233 (9.3)
	Social media posts	1523 (61.1)
	Special publicity activities	380 (15.2)
	Other	356 (14.3)
Pack-years of cigarette smoking, mean (SD)		15.56 (13.92)
**Pack-years of cigarette smoking, n (%)**		
	<20	1766 (70.6)
	≥20	734 (29.4)
**Type of smoker, n (%)**		
	Cigarette	2469 (99.1)
	Other	23 (0.9)
Age when started smoking (years), mean (SD)		17.94 (4.50)
**Age when started smoking (years), n (%)**		
	<18	1203 (48.1)
	≥18	1297 (51.9)
**Tobacco dependence, n (%)**		
	Yes	2142 (85.7)
	No	358 (14.3)
**Degree of tobacco dependence, n (%)**		
	Light	2228 (89.1)
	Medium	188 (7.5)
	Heavy	84 (3.4)
**Duration to the first cigarette after waking (minutes), n (%)**		
	≤5	854 (34.2)
	6-30	865 (34.6)
	31-60	353 (14.1)
	>60	428 (17.1)
**Hardest cigarette to give up, n (%)**		
	First one in the morning	1213 (48.5)
	Other	1287 (51.5)
**Reasons for quitting, n (%)**		
	Worried about the impact on children	986 (39.4)
	Prevention of related diseases	752 (30.1)
	Family or friends want me to stop smoking	203 (8.1)
	Other	559 (22.4)

### Effectiveness of mCessation for Smoking Cessation, Compared With Other Studies

As shown in Table 2 38.0% (950/2500) of the participants responded to the telephone interview, and the 7-day PPA rate at 6 months was 21.9% (209/953). In addition, we summarized the related real-world evidence and compared our study population, design, methods, and results with published studies. In general, the 30-day self-reported abstinence rate of telephone-interviewed participants (1971/10,340, 19.1%) [21] was higher than of message-interviewed participants (193/1470, 13% [18] and 227/6153, 3.7% [20]). The 7-day PPAs at 6 months of were 21.2% (55/259) in those <30 years old, 20.2% (79/391) in those at least 30 years old but younger than 40 years old, and 24.7% (75/303) in those at least 40 years old. Finally, the cessation rates were similar in both men and women, which should be interpreted with caution because of the small sample size.

**Table 2:** Summary of the real-world studies about message-based tobacco cessation programs.

Authors (year)	Country	Population	Sample size	Design/follow-up	Tobacco cessation program	Primary outcome	Results
Christofferson et al (2016) [18]	United States	Veterans	1470	Real-world study/message-interviewed	SmokefreeVET (message-based)	30-day self-reported abstinence at 5 weeks	193/1470 (13%)
Gopinathan et al (2018) [21]	India	General population	10,340	Real-world study/telephone-interviewed	QutiNow (message-based)	30-day self-reported abstinence at 4-6 months	1971/10,340 (19.1%)
Christofferson et al (2021) [20]	United States	Veterans	6153	Real-world study/message-interviewed	SmokefreeVET (message-based)	30-day self-reported abstinence at 6 months	227/6153 (3.7%)
Su et al (2023) [this study]	China	General population	950	Real-world study/telephone-interviewed	mCessation (message-based)	7-day point prevalence abstinence at 6 months	209/953 (21.9%); age groups: <30 years: 55/259 (21.2%); ≥30 and <40 years: 79/391 (20.2%); ≥40 years: 75/303 (24.7%); gender groups: male: 203/926 (21.9%); female: 6/27 (22.2%)

### Influencing Factors of mCessation Effectiveness

In the multivariate logistic regression analysis, the following 3 predictive factors were associated with the 7-day PPA rate at 180 days (Table 3): age ≥40 years old, being married, and heavy tobacco dependence. Compared with current smokers aged <30 years, those aged ≥40 years had a 1.77-times (95% CI 1.06-3.29; *P*=.04) higher chance of quitting smoking. A similar trend was seen in current smokers with tobacco dependence versus those without tobacco dependence (OR 1.64, 95% CI 1.08-2.51; *P*=.05). On the contrary, participants who were married or heavily dependent smokers had lower chances of quitting smoking than those who were unmarried (OR 0.57, 95% CI 0.34-0.93; *P*=.03) or light smokers (OR 0.16, 95% CI 0.02-0.98; *P*=.05), respectively. However, no significant difference in the 7-day PPA rate at 180 days was observed between current smokers with or without tobacco dependence or among different age groups when they started smoking. In addition, we explored the influencing factors of not responding to the telephone interview, and the results are shown in Table S2 in Multimedia Appendix 1. Participants who worked in a public service unit were more likely to not respond to the telephone interview (OR 1.38, 95% CI 1.03-1.83; *P*=.02) than those without jobs. The same trend was seen in subscribers reached by special publicity activities compared with those recommended by health care providers (OR 1.41, 95% CI 1.00-1.99; *P*=.04). Finally, the results of the sensitivity analysis shown in Table S3 in Multimedia Appendix 1 showed no statistical difference in baseline characteristics between respondents and nonrespondents, which indicated that not responding to the telephone interview was a random event.

**Table 3:** Adjusted odds ratios (ORs) for associations between self-reported 7-day PPA at 6 months and demographic and smoking characteristics among respondents.

Variables			Odds ratio (95% CI)		*P* value
**Age (years)**					
	<30	1		N/A^a^	
	30 to <40	1.32 (0.80-2.16)		.95	
	≥40	1.77 (1.06-3.29)		.04	
**Age when started smoking (years)**					
	<18	1		N/A	
	≥18	1.22 (0.87-1.70)		.26	
**Pack-years of cigarette smoking**					
	<20	1		N/A	
	≥20	1.07 (0.68-1.68)		.77	
**Gender**					
	Male	1		N/A	
	Female	1.02 (0.39-2.68)		.96	
**Education level**					
	Primary school or below	1		N/A	
	Middle school	1.59 (0.42-5.97)		.53	
	College/university or above	1.58 (0.43-5.83)		.53	
**Employment status**					
	Unemployed	1		N/A	
	Working in a public service unit	0.98 (0.58-1.66)		.70	
	Working in an enterprise	0.82 (0.51-1.32)		.28	
**Marital status**					
	Unmarried	1		N/A	
	Married	0.57 (0.34-0.93)		.03	
	Divorced	0.58 (0.26-1.29)		.46	
**Information source**					
	Recommended by health care providers	1		N/A	
	Social media posts	0.82 (0.48-1.39)		.19	
	Special publicity activities	0.83 (0.43-1.60)		.43	
	Other	1.28 (0.67-2.43)		.13	
**Type of smoker**					
	Cigarette	1		N/A	
	Other	1.11 (0.21-6.07)		.90	
**Tobacco dependence**					
	Yes	1		N/A	
	No	1.64 (1.08-2.51)		.02	
**Degree of tobacco dependence**					
	Light	1		N/A	
	Medium	0.96 (0.52-1.78)		.14	
	Heavy	0.16 (0.02-0.98)		.05	
**Duration to first cigarette after waking (minutes)**					
	≤5	1		N/A	
	6-30	0.96 (0.62-1.47)		.22	
	31-60	1.27 (0.74-2.16)		.49	
	>60	1.33 (0.79-2.25)		.30	
**Hardest cigarette to give up**					
	First one in the morning	1		N/A	
	Other	1.27 (0.88-1.84)		0.20	
**Reasons for quitting**					
	Worried about the impact on children	1		N/A	
	Prevention of related diseases	1.23 (0.79-1.92)		.55	
	Family or friends want me to stop smoking	0.85 (0.44-1.62)		.37	
	Other	1.23 (0.79-1.91)		.30	

^a^N/A: not applicable.

## Discussion

This study examined the real-world use of mCessation, a low-cost, text messaging, WeChat-based smoking cessation program, among smokers in the general population in China. Even though nearly three-quarters of mCessation users were younger than 40 years old, the technology did not limit the use of mCessation by older smokers: The average age was about 35 years, and the oldest user was 71 years old. Most of the mCessation users were male smokers (96.2%) and with light tobacco dependence (89.1%). For telephone interview respondents, the primary outcome, the 7-day PPA rate at 6 months, was 21.9%. Those who were older than 40 years, single, and with light tobacco dependence were significantly more likely to successfully quit smoking at 6 months than the corresponding control groups. In addition, we also found that participants who worked in a public service unit or participated in mCessation after becoming aware of it through special publicity activities were more likely to not respond to the telephone interview than the control groups.

Our data support that real-world utilization of mCessation is related with age and gender factors. The abstinence rate was higher in older people (≥40 years) than in those aged <30 years, which might be explained by more underlying diseases and greater intention to lead a healthy life in the older adults. However, consistent with previous findings in Bangladesh [27] and China [28], younger participants had higher acceptance of an online intervention than older people, which was easily understandable because young people were more familiar with digital devices, which are ubiquitous in their lives [29]. Therefore, future research should pay more attention to improving the smoking cessation rates of young people, not just the acceptability, which would further improve the effectiveness of text messaging interventions. Similarly, increasing the acceptance of mCessation by older people would have great potential benefits, based on their positive abstinence rates in the real-world settings and high rate of mobile phone use and coverage of WeChat among Chinese citizens. There is a large number of older smokers in mainland China, and a nationally representative, cross-sectional study found that the number of current smokers was higher among those aged 40 years to 69 years than among those aged 20 years to 39 years in the general population (16,705 vs 5282) [30].

In addition, the fact that nearly all mCessation participants were male could be explained by the following 2 reasons. On the one hand, the 2018 China Adult Tobacco Survey estimated the prevalence of current smoking was 50.5% in men and 2.1% in women [4]; therefore, the number of male smokers was much higher than that of female smokers. On the other hand, earlier studies in China had shown that women were less likely to accept an online approach than men [31,32], which was also reported in a study with veterans [20]. This might be because men are more task-orientated and more interested in new technology [32,33]. Moreover, inconsistent with the findings of earlier studies, we also found an association between acceptance of a text messaging intervention and educational levels, which was reflected by the high proportion of those with at least a college or university education in our study [16,34].

Among mCessation users in the real-world setting, the prevalence of tobacco dependence was higher than in our previous cross-sectional study in the general population [30]. This suggests that tobacco dependence was associated with an individual’s acceptance of the text messaging intervention, because smoking dependence was the main reason for challenges with stopping smoking [35]. Consistent with the findings of a cluster RCT with Chinese adolescents [36], we believed that a mobile phone text messaging intervention could encourage those dependent on cigarettes to quit smoking, especially for those who were mostly light smokers. The proportions of individuals with a light tobacco dependence were about 50% in the general population [30] but nearly 90% in mCessation participants, which indicates that these lightly dependent populations have a higher willingness to quit smoking than those with a moderate or severe dependence. For those with a severe tobacco dependence, it is difficult to quit smoking on their own initiative, because they tend to have a complex and severe condition involving physiological, psychological, and behavioral processes, which is considered a mental disorder by the ICD [37].

We first reported that, among mCessation telephone respondents, the real-world 7-day PPA at 6 months was 21.9%, which is higher than in previously reported RCTs (12.1%-17.7%) [12,13]. We believe the difference in the abstinence rates between our real-world study and previous RCTs was likely due to the underlying population differences: The proportion of individuals with a light tobacco dependence (89.1%) was higher than in RCT studies in Hong Kong (51.3%) [13] and Norway (29.4%) [12]. A cluster RCT conducted in the Chinese population supported that a mobile phone text messaging intervention could inhibit cigarette dependence in adolescent smokers who were mostly light smokers [36]. In addition, the 21.9% abstinence rate in our study was similar to that in a study in India (19.1%) [21] but higher than those in 2 studies conducted with veterans (13% in 2016 [18]; 3.7% in 2021 [20]). This might be due to differences in follow-up methods (telephone interviews vs message interviews) and the targeted population (general population vs veterans).

In the multivariate analysis, we also found that those with a heavy dependence were less likely to quit smoking. On the contrary, older adult or single smokers had higher chances of stopping smoking, which may be because older adults prefer to make behavioral changes due to high health awareness, while single individuals pay more attention to giving the impression of a healthy lifestyle to find a partner [38]. Notably, a follow-up telephone interview to determine abstinence rates was of great importance to evaluate the effectiveness of a text messaging intervention in the real world. Published real-world data had shown quite low response rates of subscribers to text messaging programs [20,21], which could lead to bias in smoking cessation rates. For example, with the primary outcome of self-reported 30-day PPA at 6 months, the abstinence rate determined by telephone interview was 19% in a study in India [21] but only 3.7% in a study with veterans without a telephone interview [20]. Therefore, we also explored the influencing factors of nonresponse to the follow-up telephone interview, and the results suggested that, to improve the response rates to telephone interviews, we should add more interventions that target those working in public service units and who found out about mCessation through special publicity activities.

The strengths of this real-world study were that our target population included current smokers in the general population and answering the smoking questions in mCessation was mandatory, which minimized the bias related to the populations generally included in previous studies. For example, some participants who enrolled in research conducted with veterans [18,20] or pregnant individuals [19] were likely not veterans or pregnant women, respectively; even nonsmokers subscribed to the smoking cessation program in the study in India. More importantly, a long-term abstinence rate, the 7-day PPA rate at 6 months, was selected as the primary outcome; this was necessary to evaluate the smoking cessation intervention since tobacco dependence is a chronic, relapsing condition. However, there were still some limitations. First, data for the primary outcome of the abstinence rate were self-reported and collected by telephone interview, which may lead to information bias affecting our results. Therefore, sensitivity analysis could increase the robustness of our results. Second, response rates to the telephone follow-up were lower than 40%, indicating that strategies to improve health awareness and public cooperation were critical to the evaluation of the effectiveness of the smoking cessation program in the real-world setting. Finally, our data collection did not include receipt of additional tobacco cessation care such as the use of smoking cessation medications. It was possible that additional interventions received by the subscribers may have influenced abstinence rates, which would lead to overestimation of our results in this study.

Analysis of mCessation provided important information on the demographics of real-world users, suggesting that the program was more likely to serve a population that has the following characteristics: younger age, men, and light degree of tobacco dependence. The main finding of this analysis was that mCessation could help 1 in every 5 smokers aged 18 years to 67 years quit smoking, but the real-world effectiveness was less significant among young, married, and heavily dependent smokers compared with the control groups. Tobacco control is of great public health significance because tobacco use is one of the main risk factors for noncommunicable diseases (cancer, cardiovascular disease, and respiratory disease), which are a major health threat globally [37]. Thus, a low-cost, efficacious, and easily and publicly accessible smoking cessation modality such as text messages or a mobile app is suitable in China or LMICs. In the future, more studies of how to improve the popularity of mobile cessation programs and their effectiveness in insensitive populations are needed.

## Appendix

Multimedia Appendix 1 Supplemental tables.

## Abbreviations

FTND: Fagerström Test for Nicotine Dependence
ICD-10: International Classification of Diseases
LMICs: low- and middle-income countries
OR: odds ratio
PPA: point prevalence abstinence
RCT: randomized controlled trial
WHO: World Health Organization

## Data Availability

All data in this study are not publicly available but could be accessed by asking the corresponding author.
