# Supplementary material for: Utilization and Effectiveness of a Message-Based Tobacco Cessation Program (mCessation) in the Chinese General Population: Longitudinal, Real-world Study
Source: J Med Internet Res. 2023 May 2;25:e44840. doi: 10.2196/44840 (PMC10189622; doi:10.2196/44840)
Supplement: Multimedia Appendix 1 [file jmir_v25i1e44840_app1.docx]

**Table S1.** Items and scoring of the Fagerström Test for Nicotine Dependence.

| **Question** | **Answers** | **Points** |
| --- | --- | --- |
| 1. How soon after you wake up do you smoke your first cigarette? | Within 5 minutes | 3 |
|  | 6-30 minutes | 2 |
|  | 31-60 minutes | 1 |
|  | After 60 minutes | 0 |
| 2. Do you find it difficult to refrain from smoking in places where it is forbidden, e.g., in church, at the library, in the cinema, etc.? | Yes | 1 |
|  | No | 0 |
| 3. Which cigarette would you most hate to give up? | The first one in the morning | 1 |
|  | Any other | 0 |
| 4. How many cigarettes/day do you smoke? | 10 or less | 0 |
|  | 11-20 | 1 |
|  | 21-30 | 2 |
|  | 31 or more | 3 |
| 5. Do you smoke more frequently during the first hours after waking than during the rest of the day? | Yes | 1 |
|  | No | 0 |
| 6. Do you smoke if you are so ill that you are in bed most of the day? | Yes | 1 |
|  | No | 0 |

**Table S2.** Multivariate logistical regression analysis of the predictors of the nonresponse rate.

| **Variables** | OR | 95%CI | P |
| --- | --- | --- | --- |
| **Age** | 1.00 | 0.98, 1.01 | 0.50 |
| **Age at starting smoking** | 1.00 | 0.98, 1.02 | 0.70 |
| **Pack-years of cigarette smoking** | 1.00 | 0.99, 1.01 | 0.70 |
| **Gender** |  |  |  |
| Male | 1.00 |  |  |
| Female | 1.45 | 0.91, 2.37 | 0.12 |
| **Education level** |  |  |  |
| Primary school or below | 1.00 |  |  |
| Middle school | 1.05 | 0.57, 1.88 | 0.90 |
| College/university or above | 0.82 | 0.45, 1.46 | 0.50 |
| **Employment status** |  |  |  |
| Unemployed | 1.00 |  |  |
| Working in public service unit | 1.38 | 1.03, 1.83 | 0.02 |
| Working in enterprises | 1.28 | 1.00, 1.65 | 0.05 |
| **Marriage status** |  |  |  |
| Unmarried | 1.00 |  |  |
| Married | 0.87 | 0.68, 1.11 | 0.30 |
| Divorced | 0.78 | 0.52, 1.17 | 0.20 |
| **Information Source** |  |  |  |
| Recommended by health care providers | 1.00 |  |  |
| Social media propaganda | 1.16 | 0.87, 1.55 | 0.30 |
| Special publicity activities | 1.41 | 1.01, 1.99 | 0.04 |
| Other | 1.41 | 0.99, 2.00 | 0.06 |
| **Type of smoker** |  |  |  |
| Cigarette | 1.00 |  |  |
| Other | 0.88 | 0.37, 2.15 | 0.80 |
| **Tobacco dependence** |  |  |  |
| Yes | 1.00 |  |  |
| No | 1.08 | 0.91, 1.28 | 0.40 |
| **Degree of tobacco dependence** |  |  |  |
| Light | 1.00 |  |  |
| Medium | 0.75 | 0.55, 1.03 | 0.07 |
| Heavy | 1.16 | 0.70, 1.98 | 0.60 |

**Table S3.** Differences in baseline characteristics and smoking profiles of respondents (n=953) versus nonrespondents (n=1547).

| Variables | Respondents, n (%) | Nonrespondents, n (%) | *P* value |
| --- | --- | --- | --- |
| **Age (years)** | | | .07 |
| <30 | 259 (27.2) | 486 (31.4) |  |
| ≥30, <40 | 391 (41.0) | 611 (39.5) |  |
| ≥40 | 303 (31.8) | 449 (29.0) |  |
| **Gender** | | | .08 |
| Male | 925 (97.2) | 1481 (95.7) |  |
| Female | 27 (2.8) | 66 (4.3) |  |
| **Education level** | | | .06 |
| Primary school or below | 20 (2.1) | 39 (2.5) |  |
| Middle school | 233 (24.4) | 445 (28.8) |  |
| College/university or above | 700 (73.5) | 1061 (68.7) |  |
| **Employment status** | | | .73 |
| Unemployed | 141 (14.8) | 211 (13.7) |  |
| Working in public service unit | 264 (27.7) | 433 (28.0) |  |
| Working in enterprises | 548 (57.5) | 900 (58.3) |  |
| **Marital status** | | | .09 |
| Unmarried | 223 (23.4) | 422 (27.3) |  |
| Married | 674 (70.7) | 1033 (66.9) |  |
| Divorced | 56 (5.9) | 88 (5.7) |  |
| **Information source** | | | .18 |
| Recommended by health care providers | 98 (10.3) | 135 (8.8) |  |
| Social media propaganda | 595 (62.6) | 928 (60.2) |  |
| Special publicity activities | 134 (14.1) | 246 (16.0) |  |
| Other | 124 (13.0) | 232 (15.1) |  |
| **Pack-years of cigarette smoking, mean (SD)** | | | .25 |
| < 20 pack-years | 661 (69.4) | 1104 (71.4) |  |
| ≥ 20 pack-years | 292 (30.6) | 442 (28.6) |  |
| **Type of smoker** | | | >.99 |
| Cigarette | 940 (99.1) | 1529 (99.1) |  |
| Other | 9 (0.9) | 14 (0.9) |  |
| **Age at starting smoking (years), mean (SD)** | | | .41 |
| <18 years | 448 (47.0) | 754 (48.8) |  |
| ≥ 18 years | 505 (53.0) | 792 (51.2) |  |
| **Tobacco dependence** | | | .55 |
| Yes | 530 (55.6) | 840 (54.3) |  |
| No | 423 (44.4) | 707 (45.7) |  |
| **Degree of tobacco dependence** | | | .18 |
| Light | 847 (88.9) | 1381 (89.3) |  |
| Medium | 80 (8.4) | 108 (7.0) |  |
| Heavy | 26 (2.7) | 58 (3.7) |  |
